# Biology of p53 protein isoforms and their significance in hematological malignancies

**DOI:** 10.3389/fonc.2026.1854189

**Published:** 2026-06-02

**Authors:** Anna Maria Janik, Zuzanna Tracz-Gaszewska, Irena Misiewicz-Krzemińska, Krzysztof Jamroziak

**Affiliations:** 1 Department of Hematology, Transplantation and Internal Medicine of Medical University of Warsaw, Warsaw, Poland; 2 Department of Experimental Hematology, Institute of Hematology and Transfusion Medicine, Warsaw, Poland

**Keywords:** cancer progression, chemosensitivity, hematological malignancies, isoform detection, p53 isoforms, prognostic biomarkers

## Abstract

The tumor suppressor gene *TP53* produces multiple p53 protein isoforms through alternative promoter usage, messenger RNA splicing, and alternative translation initiation. These isoforms can differentially shape canonical p53 outputs, including cell-cycle arrest, senescence, apoptosis, and DNA-damage responses, and are increasingly recognized as context-dependent regulators of tumor progression and treatment response. However, their roles in hematological malignancies remain incompletely characterized. This review summarizes the structure, biogenesis, and functional consequences of p53 isoforms and integrates available evidence on their expression across acute leukemias, multiple myeloma, chronic lymphocytic leukemia, and selected non-Hodgkin lymphomas. We highlight reported differences between diagnostic and relapse specimens, associations with cytogenetic risk categories, and interactions with TP53 mutation backgrounds. Available data suggest that N-terminally truncated isoforms (Δ40p53, Δ133p53/Δ160p53) and C-terminally spliced variants (p53β/γ) can modulate tetramer composition and downstream transcriptional programs, thereby influencing apoptosis, senescence, and DNA repair. However, the clinical significance of these patterns in hematological malignancies remains incompletely defined because the evidence is derived from relatively small and methodologically heterogeneous studies. More standardized assays and prospective validation in larger, clinically annotated cohorts are needed before p53 isoform profiling can be incorporated into routine risk stratification or therapeutic decision-making. We also discuss methodological advances and limitations in isoform detection at transcript and protein levels, including isoform-specific quantitative reverse-transcription polymerase chain reaction, antibody panels, capillary nanoimmunoassays, and targeted liquid chromatography–tandem mass spectrometry. Comprehensive profiling of p53 isoforms may eventually help refine biological classification and risk assessment, but its clinical utility will depend on assay standardization and prospective validation.

## 1 Introduction

*TP53* is a tumor suppressor gene located on the short arm of human chromosome 17 (17p13.1) and comprises 11 exons, 10 introns, and 393 amino acid residues (1–3). It encodes a transcription factor p53 protein that is established as a „guardian of the genome” (4). Data show that *TP53* is the most frequently mutated gene in human cancers (5). Somatic mutations in the *TP53* gene are among the most common alterations (6), while germline mutations confer inherited susceptibility to cancer (7). *TP53* mutations are found in approximately 50% of human cancers, reaching 90% in small cell lung cancer (8, 9). Despite the wide diversity of genes implicated in tumorigenesis, the *TP53* mutation is most frequently associated with a poor prognostic outcome across all cancer types (10, 11).

In hematological cancers, *TP53* mutations are found much less frequently, specifically in 10-30% of patients (12, 13). Depending on the type of hematologic malignancy, *TP53* alterations are found in 5-10% of patients with multiple myeloma (MM) (14), 3-12% with acute myeloid leukemia (AML) (15–19), 16% with acute lymphocytic leukemia (ALL), 6% with myelodysplastic syndrome (MDS) (10), 7%-20% with chronic lymphocytic leukemia (CLL) (10, 17), 20-30% of chronic myeloid leukemia (CML) blast phases, 15% of Burkitt lymphomas (13), 9-11% of Hodgkin lymphoma (20, 21), and in 30% of high-grade B-cell non- Hodgkin lymphoma cases (13). Moreover, the incidence is increased in case of refractory or relapsed disease (22). Deletions of all or part of the short arm of chromosome 17 (17p deletion, del17p) represent a recurrent cytogenetic abnormality detected across multiple human malignancies (23). In hematological tumors, 17p deletions are generally infrequent at diagnosis but become more common in high−risk and relapsed disease, particularly in CLL and MM (23). Furthermore, they correlate with aggressive disease progression, treatment resistance, and short survival (24, 25).

*TP53* splice variants were first discovered in the 1980s (26, 27), however, their existence across various species and their significance both biologically and clinically were only confirmed about 15 years thereafter (28). Studies have demonstrated that isoform expression can occur independently of *TP53* mutation status (29–31). Moreover, the mutation status alone is insufficient to predict the course of cancer progression accurately (29). p53 isoforms were noted to show contradictory effects, as they can promote both cell growth and cell death, depending on the specific cellular environment (28). The disruption of p53 isoform co-expression can change, but not eliminate, the expected response of p53. Interestingly, this change may promote cancer development while also increasing sensitivity to a specific cancer treatment (28, 32–35). Among the numerous factors that govern a cell’s progression toward malignancy, the expression levels of p53 isoforms play a pivotal role.

## 2 P53 protein and its isoforms

### 2.1 Cellular function of p53

The p53 protein functions as a transcription factor that requires homo-tetramerization to perform its canonical function (36). It is typically categorized into three main regions that can be divided into five domains: the N-amino-terminal (N-terminal) region which contains transactivation domains (TAD1, TAD2) that regulates the transcription of target genes and proline-rich domain (PRD); centrally located core region containing DNA binding domain (DBD) that binds to specific DNA sequences, and the carboxy-terminal (C-terminal) region that is divided into tetramerization/oligomerization (OD) domain and regulatory domain (CTD), both essential for the regulation of p53 activity (1, 37–42). In healthy cells, the level of p53 is usually low (43), and it is characterized by an unstable conformation and a short half-life due to persistent degradation via ubiquitination, mediated by its negative regulator, mouse double minute 2 (MDM2) (44–47). Under environmental stress, ubiquitination is inhibited, and intracellular p53 levels increase immediately. Then, p53 gets stabilized and activated through post-translational modifications such as phosphorylation, acetylation, and methylation, and accumulates in the cell (48–51). Active p53 translocates to the cell nucleus, where it functions as a transcription factor.

The p53 pathway acts as a critical cellular defense mechanism against a wide range of stress signals, including oxidative stress, endoplasmic reticulum stress, oncogene activation, DNA and RNA damage, cell-cell contact disruption, cytokine secretion, telomere shortening, hypoxia, nutrient deprivation, and viral infection (42, 52–54). Known as a tumor suppressor protein, p53 primarily functions as a transcription factor that regulates the expression of approximately 500 target genes to counteract destructive factors that affect the cell (36). Through these genes, p53 orchestrates diverse cellular responses, including cell cycle arrest, DNA repair, apoptosis, senescence, and alterations in cell adhesion, migration, and motility. These processes not only maintain genomic stability but also prevent malignant transformation by suppressing tumor growth and progression. In addition to direct transcriptional activation or repression of effector genes, p53 influences multiple signaling pathways indirectly, integrating cellular stress signals to modulate metabolism, autophagy, and immune responses (4, 32, 52, 55, 56). Furthermore, p53 activity is tightly regulated by post-translational modifications and interactions with various proteins, enabling a finely tuned response tailored to the type and severity of stress (47). This multifaceted role of p53 underscores its importance in safeguarding cellular integrity and preventing carcinogenesis under both genotoxic and non-genotoxic stress.

Wild- type p53 (WTp53) and mutated p53 (mutp53) differ in stability in the cellular environment and can perform entirely different functions (57, 58). The WTp53 is essential in regulating the transcription of numerous target genes. It plays a pivotal role in many critical biological processes (36). In contrast, mutated p53 (mutp53) may either lose tumor suppressor function, thereby promoting invasion, proliferation, and cell survival and contributing to both cancer progression and metastasis, or gain new functions that influence other genes and promote tumorigenesis (59–63).

Comprehensive sequencing shows that the majority of *TP53* alterations are single-base substitutions rather than large deletions or complex rearrangements (64). Within the coding region, these substitutions are predominantly missense mutations (>80%) that cluster between codons 125–300, corresponding to the DNA-binding domain (6), especially in Arg175, Gly245, Arg248, Arg249, Arg273, and Arg282 residues (65, 66). Among myeloid cancers, these hotspot mutations represent about 35% of all TP53 missense mutations (12). The formation of missense mutations can be categorized into three different mechanisms: 1) loss of function (LOF) of transcriptional regulatory activity toward p53-responsive genes, 2) dominant negative effect (DNE) of the WTp53 activity through the formation of heterotetrameric complexes and 3) gain-of-function (GOF) properties associated with enhanced oncogenic activity (67–70).

### 2.2 P53 protein isoforms - the definition and mechanisms of formation

**Figure 1:**
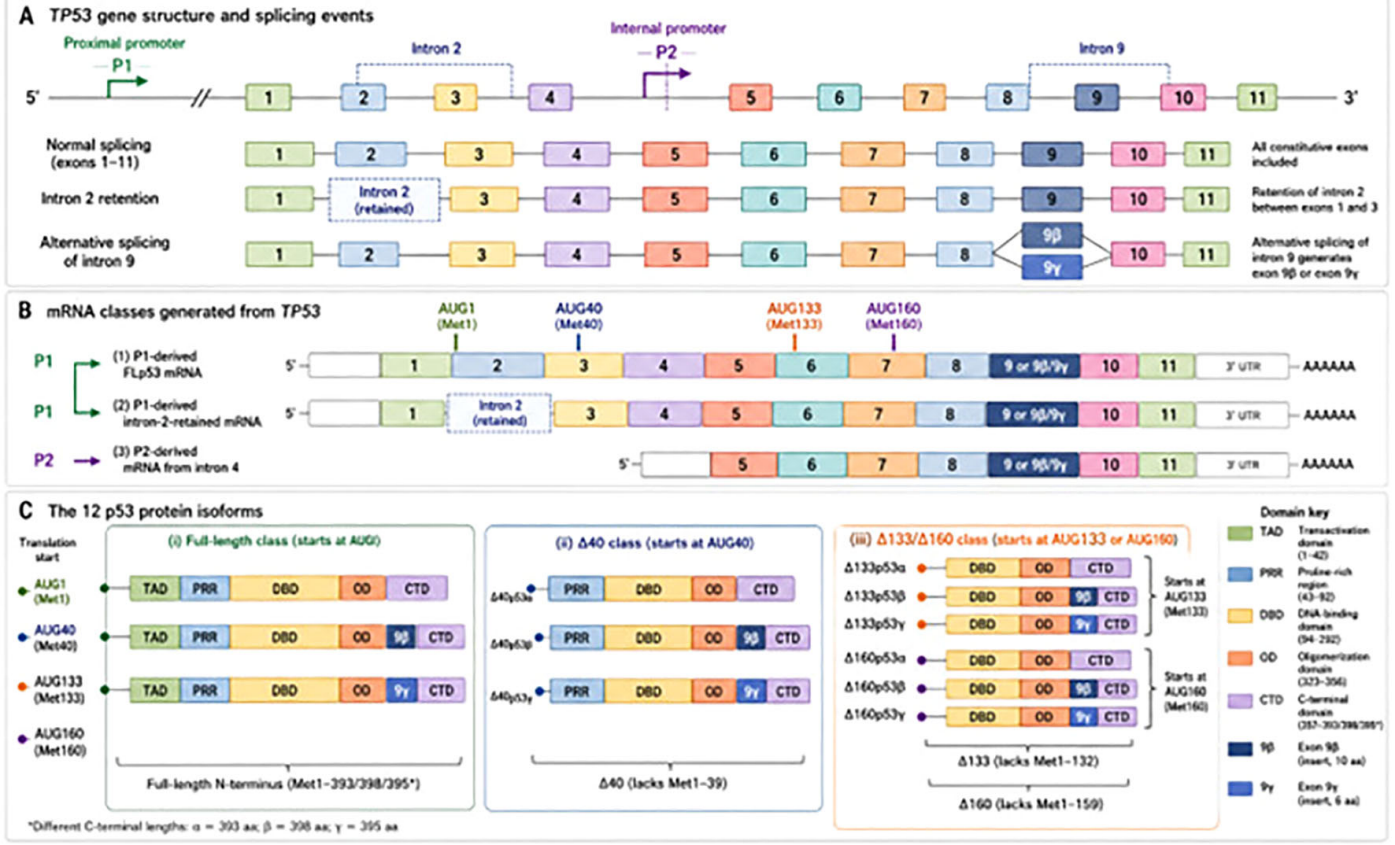
The structure of *TP53* gene and mechanisms of p53 isoforms formation. **(A)** TP53 gene structure and splicing events. The human TP53 gene consists of 11 exons (numbered boxes) spanning from the 5’ to 3’ end. Alternative splicing mechanisms include normal splicing (exons 1–11), intron 2 retention, and alternative splicing of intron 9. The proximal promoter P1 is located upstream of exon 1, while the internal promoter P2 is positioned within intron 4. Intron 9 undergoes alternative splicing to generate exons 9β and 9γ, which contain premature stop codons. **(B)** mRNA classes generated from TP53. The TP53 gene produces multiple mRNA variants through alternative promoter usage and splicing. P1-driven transcription yields full-length p53 (FLp53) mRNA and Δ40p53-encoded mRNAs (with or without intron 2 retention). P2-driven transcription generates Δ133- and Δ160-truncated mRNAs initiating from intron 4. Alternative start codons are indicated: AUG1 (Met1) for FLp53, AUG40 (Met40) for Δ40p53, AUG133 (Met133) for Δ133p53, and AUG160 (Met160) for Δ160p53. All mRNAs contain poly **(A)** tails at the 3’ UTR. **(C)** Protein isoforms and functional domains. Translation from different start codons produces p53 isoforms with distinct N-terminal structures. Functional domains are color-coded: TAD (transactivation domain, yellow), PRR (proline-rich region, purple), DBD (DNA-binding domain, orange), OD (oligomerization domain, cyan), and CTD (C-terminal domain, pink/purple variants). Full-length p53 isoforms (FLp53α/β/γ) span 393, 356, and 359 amino acids, respectively. Δ40 isoforms (Δ40p53α/β/γ) lack Met1–39 and range from 354 to 320 amino acids. Δ133 and Δ160 isoforms lack the entire TAD and PRR domains, with lengths of 261 and 233 amino acids (α variants), respectively. C-terminal variants (α, β, γ) differ in the CTD structure and length based on alternative splicing of intron 9. Domain boundaries and isoform-specific structural features are indicated. TAD, transactivation domain; PRR, proline-rich region; DBD, DNA-binding domain; OD, oligomerization domain; CTD, C-terminal domain; UTR, untranslated region. Different C-terminal lengths: α = 393 aa, β = 356 aa, γ = 359 aa.

**Figure 2:**
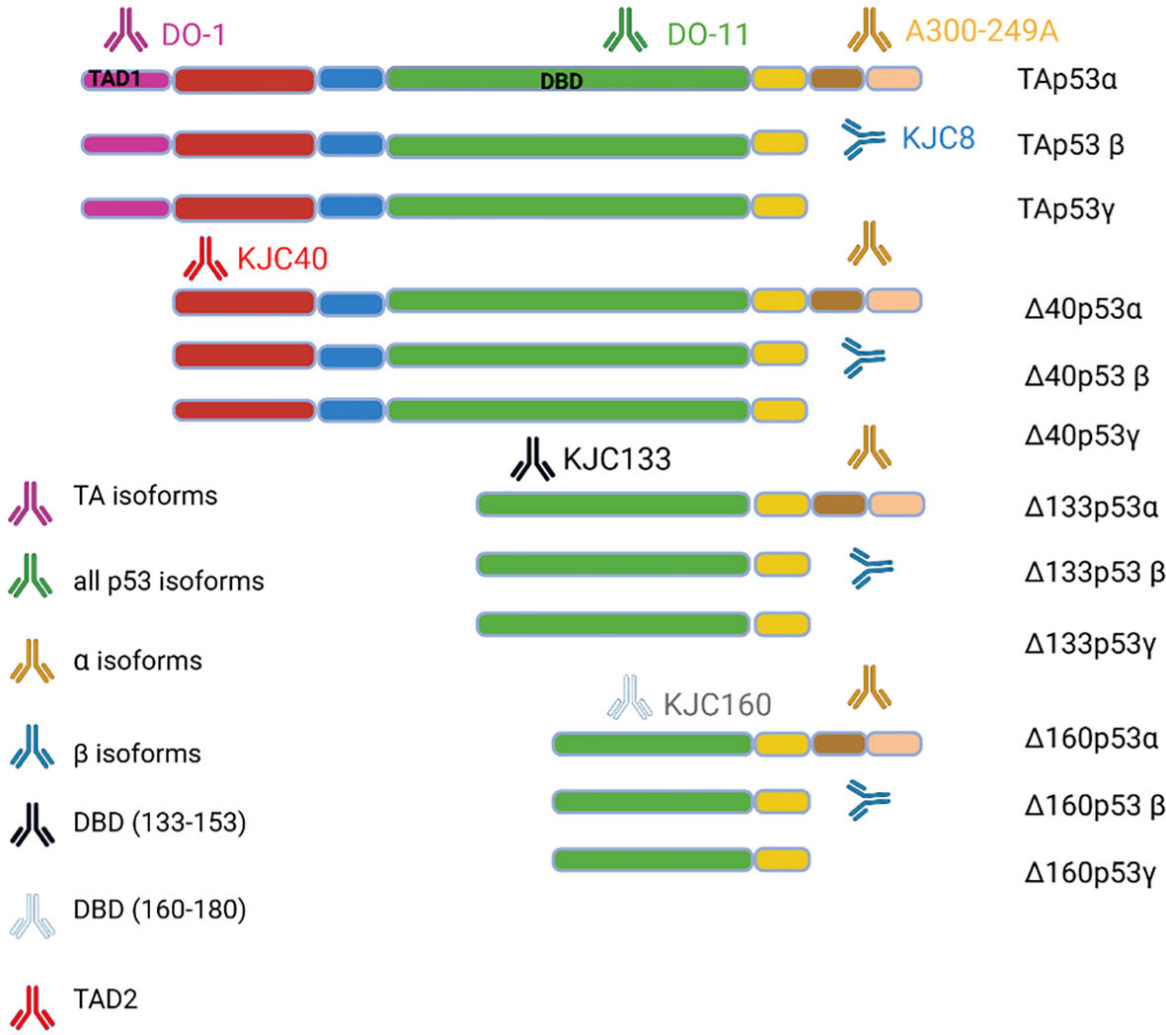
Schematic composition of p53 isoforms. Each color represents distinct domain: pink - transactivation domain 1 (TAD1), red - transactivation domain 2 (TAD2), blue - proline-rich domain (PRD), green - DNA-binding domain (DBD), yellow - nuclear localization domain (NLS), brown - oligomerization domain (OD), and salmon pink - C-terminal regulatory domain (CTD). The antibodies reflect the site where they bind, and the region detected.

In 2005, Bourdon et al. identified an alternative promoter in the *TP53* gene, leading to the discovery and characterization of p53 isoforms (71). Currently, it is known that due to alternative splicing and the usage of alternative promoters, *TP53* expresses at least twelve isoforms of p53 protein: p53α (also named full-length p53=FLp53, canonical p53, WTp53, TAp53α), p53β (or p53i9), p53γ, Δ40p53α (or ΔNp53, p44 or p47), Δ40p53β, Δ40p53γ, Δ133p53α, Δ133p53β, Δ133p53γ, Δ160p53α, Δ160p53β, and Δ160p53γ. These isoforms, generated from nine different mRNA transcripts, exhibit overlapping or distinct activities (72–75). Based on the molecular mechanisms involved in their formation, the isoforms can be classified into subclasses and variants, including α, β, γ, transactivation domain, and the Δ40 and Δ133/Δ160 variants (72). One mechanism for generating p53 isoforms is the selection of alternative promoters (proximal P1 and internal P2 in intron 4) (74). In addition, depending on the cell type, p53 isoforms can arise from the use of alternative initiation codons within the same mRNA. Specifically, transcription from promoter 1 generates an mRNA that encodes full-length p53 (FLp53) isoforms via translation initiation at the canonical start codon (AUG1). When intron 2 that contains an in-frame stop codon is retained through alternative splicing, it causes premature translation termination of the FLp53 protein, allowing for the initiation of the Δ40p53 isoform from a downstream start codon AUG40. P2 is responsible for the formation of the Δ133 and Δ160 isoforms using different start codons AUG133 and AUG160, respectively (74, 76–79). Moreover, the C-terminal isoforms are generated when intron 9 is alternatively spliced, producing the 9β and 9γ exons, which contain stop codons that truncate the protein, preventing the inclusion of exons 10 and 11 (74, 75). The structure of *TP53* gene and mechanisms of p53 isoforms formation are shown in **Figure 1**. P53 isoforms exhibit distinct structural features based on their protein domains. FLp53 isoforms contain TAD1 and TAD2, PRD, DBD, and OD which includes a nuclear localization domain (NLS) and additional five regions preserved during evolution (74, 80, 81). Whereas Δ40p53 isoforms lack the first transactivation domain but retain TAD2 and PRD domains (75, 80). Finally, Δ133p53 and Δ160p53 isoforms lack both TAD, PRD domain, and a part of DBD, but they retain complete OD (75, 77). The central DBD is responsible for recognizing and binding to specific DNA sequences. Thus, it is crucial for p53’s role as a transcription factor in regulating gene expression and is present in FLp53 and Δ40p53 isoforms (28). However, Δ133p53 isoforms lack a part of the first conserved cysteine box of the DBD (28), while Δ160p53 isoforms lack the cysteine box, thus significantly compromising DNA binding (75). At the C-terminal region, α isoforms contain the full oligomerization domain (OD) and the complete negative regulatory domain (74). In contrast, β and γ isoforms exhibit partial deletion of the OD and lack the entire negative regulatory domain, which is replaced by unique sequences of 10 and 15 amino acids, respectively (74, 80). The structure of p53 protein isoforms is shown in **Figure 2**.

### 2.3 P53 isoforms - selected biological functions

The biological functions of p53 protein isoforms are diverse and complex. They play crucial roles in various cellular processes. Moreover, they can co-exist and work together, cooperating or antagonizing each other. We should view p53 not as a single molecule, but as a dynamic group of oligomers composed of different isoforms. The specificity of the p53 response is dictated by the combined activity of these diverse complexes, each having its own transcriptional signature. Consequently, modulating the isoform landscape using small molecules allows for the fine-tuning of cell fate, explaining how the same stimulus can trigger either cell repair or programmed death (82).FLp53α is the primary isoform essential for maintaining genomic stability and acting as a critical barrier against tumor formation; however, it is observed that FLp53α expression is always accompanied by other p53 isoforms (13). Its C-terminally altered or N-terminally truncated forms may act in different or opposite ways, for example they may play additional role in inducing resistance to the anti-cancer therapy (75).

#### 2.3.1 Regulation of cell cycle and senescence

The p53 protein isoforms play diverse and sometimes opposing roles in regulating cell cycle arrest and cellular senescence in response to various cellular stresses. Several isoforms, including Δ40p53, Δ133p53, and p53β, influence senescence by modulating the expression of p53 target genes such as p21, miR-34a, and 14-3-3σ (83). Δ40p53 promotes cell cycle arrest and senescence through activation of 14-3-3σ and p21, particularly under stress conditions such as endoplasmic reticulum stress (84, 85). Moreover, p53β enhances senescence by forming complexes with p53α consequently leading to increased expression of p21 (86). In contrast, Δ133p53 has dual role depending on context. It can either induce senescence under oxidative stress (87) or suppress this process by preventing p53α from activating p21 and miR-34, both of which are the key pro-senescence mediators (86, 88, 89). Overall, different p53 isoforms regulate the balance between cell cycle arrest and senescence by selectively activating or repressing p53 target genes.

#### 2.3.2 Modulation of apoptosis

Another crucial function of p53 isoforms is managing apoptosis, primarily in response to DNA damage, such as from genotoxic stress or ionizing radiation. Whereas p53α is a very strong inducer of apoptosis, other isoforms enhance or inhibit this process (71, 74, 90). It can either trigger programmed cell death by activating pro-apoptotic proteins like BAX, NOXA, PUMA, Scotin, FAS or by inhibiting anti-apoptotic proteins such as BCL-2 and BCL-XL (91–93). It has been reported that the expression of Δ133p53α supports cell survival after stress and protects cells from apoptosis mainly by forming protein complex with FLp53 that antagonizes its activity but also by modulating the expression of p53 target genes such as MDM2, cyclin G, p21, and BCL-XL (94–96). Moreover, Δ133p53β can influence programmed cell death by suppressing the function of the anti-apoptotic protein RhoB (97). In contrast, Netrin-1, another protein that prevents apoptosis, can be upregulated by Δ40p53 (98). When Δ40p53 is expressed at levels equal to or lower than p53α, it promotes the expression of BAX and PIDD, which in turn triggers apoptosis (99, 100).

The isoforms of the p53 protein influence apoptosis primarily through interactions with p53α, which alter p53 homo-tetramer composition (83), thereby representing the transcriptionally active form of p53 (63, 76). P53 protein isoforms that possess functional C-terminal OD form homo-tetramers through a stepwise oligomerization process mediated by this domain. Initially, p53 monomers associate to form dimers, and then these two dimers interact to generate a stable homo-tetramer form that is capable of binding DNA and regulating transcription. Differences among p53 isoforms, such as N- or C-terminal truncations, can influence tetramer stability and transcriptional efficiency by altering these protein–protein interactions (76, 101). For example, the formation of a heterocomplex between p53β and p53α enhances p53 transcriptional activity at the BAX promoter, leading to increased apoptosis induction (71, 83). It is said that the formation of hetero-oligomers composed of p53 with Δ133p53α and/or Δ160p53α may function as a regulatory “brake” on the formation of transcriptionally active p53 homo-tetramers. This mechanism could help prevent cells from entering senescence or from undergoing cell death too early, thereby giving them time window to recover from DNA damage (102). This shows that different p53 isoforms modulate apoptosis by altering FLp53α transcriptional activity, oligomerization, stability, and DNA binding, producing context-dependent pro- or anti-apoptotic outcomes. Isoform ratios and cellular context determine whether cells undergo apoptosis or favor repair and survival.

#### 2.3.3 Response to DNA damage and DNA repair

p53 isoforms are also strongly involved in DNA damage response and repair (32). DNA damage results in a rise in the half-life and translation of p53 (103–106), allowing the tumor suppressor to drive suitable cellular reactions via the regulation of transcription and interactions with proteins (104, 106). DNA double-strand breaks (DSB) caused, for example, by ionizing radiation, reactive oxygen species, or chemotherapeutic agents, are one of the most catastrophic and genotoxic damages leading to cancer formation (107). It has been reported that Δ133p53 plays an important role in stimulating DSB repair (108) by activating DNA repairing genes such as RAD51, RAD52, and LIG4 (83, 109–111). In conclusion, FLp53 is the core coordinator of DNA repair, and its isoforms act as modulators that regulate the strength, speed, and direction of the DNA damage response.

#### 2.3.4 Involvement in cancer progression and therapy response

It is well known that the expression patterns of p53 isoforms play a significant role in cancer progression and prognosis (34). Depending on the type of cancer, overexpression of several specific isoforms slow down tumor progression, including Δ133p53 in high grade serous ovarian cancer (112), Δ40p53 in mucinous ovarian cancer (113) and hepatocellular carcinoma (85), p53β in renal cell carcinoma (114, 115), while in others cases it may accelerate it, including Δ160p53 and Δ133p53β in melanoma (116, 117), Δ133p53 in colorectal cancer (118), Δ133p53β in prostate cancer (119) and glioblastoma (120), Δ133p53 in lung cancer (121), Δ40p53 in endometrial carcinoma (122), and p53Ƴ in uterine serous carcinoma (29, 112). In addition, in colorectal cancer, researchers suggest that the ratio of p53β to Δ133p53α may serve as a predictor for the transition from adenoma to carcinoma (86). Moreover, it has been observed that p53 isoform expression can potentially predict therapeutic response (123). The most apparent results were published for breast cancer, in which it was revealed that p53 isoforms expression is strongly related to prognosis and alters therapeutic outcomes. Scientists showed that high Δ40p53:p53α ratio correlates with reduced disease-free survival. Overexpression of Δ40p53 weakens cellular sensitivity to doxorubicin, whereas Δ40p53 knockdown has the opposite effect (34). These studies suggest that p53 isoform expression can be used as a potential biomarker of both therapy response and risk of progression in many types of neoplasms, however further investigation is required to determine the utility of p53 isoforms expression in this regard.

In summary, specific isoforms (Δ113/Δ133, Δ40, p53β, Δ246 and others) switch p53 signaling between apoptosis, senescence and repair, with clear implications for therapy response. Moreover, dysregulation between their expression can contribute to therapy resistance and treatment failure. The study of isoforms enhances our understanding of *TP53* in cancer research and indicates that assessing and potentially adjusting certain p53 isoforms might better forecast response to radiation therapy and chemotherapy. However, practical use necessitates further focused investigation (34, 75, 102, 124).

### 2.4 p53 isoforms in context of wild-type and mutated *TP53*

Monoallelic *TP53* mutation reshapes the p53 network, a process potentially influenced by the expression and interaction of various p53 isoforms. In this heterozygous setting, the wild-type *TP53* allele retains the capacity to encode the canonical full-length protein. It can also produce alternative isoforms—such as Δ133p53 and Δ160p53—generated through distinct regulatory mechanisms, including alternative promoter usage and alternative initiation of translation, within the TP53 locus (75, 83, 95). Importantly, the mutated allele retains these exact same regulatory mechanisms, meaning that cells can additionally co-express mutated variants of these short isoforms (e.g., mutant Δ133p53). However, because current functional studies and antibody-based detection methods predominantly focus on isoforms derived from the wild-type sequence, the specific biological roles and tetramerization dynamics of mutant-derived short isoforms remain a significant knowledge gap in the field. Within this framework, the schematic highlights that these isoforms do not act in isolation but dynamically integrate with wild-type and mutant p53 to modulate the balance between tumor suppression and oncogenic signaling. N-terminally truncated isoforms can behave as modulators of p53, either attenuating wild-type p53-dependent transcription or amplifying mutant p53-associated oncogenic signaling in a highly context-dependent manner (125). Current evidence indicates that Δ133p53 and Δ160p53 retain the oligomerization domain and readily co-assemble with full-length p53, forming hetero-tetramers whose function depends on their stoichiometry relative to wild-type subunits. When present in excess, these isoforms can exert dominant-negative effects by disrupting full-length p53 DNA binding and transcriptional activity, and by promoting co-aggregation that alters p53 conformation, cellular distribution, and stability (102, 126). In some contexts, these same isoforms display gain-of-function properties, including promotion of cell survival, migration, and invasion, particularly in tumors harboring *TP53* mutations (125).

The functional impact of the p53 network is governed by the relative ratios of its components rather than their absolute levels, because p53 tetramers incorporate a stochastic mix of subunits, including full-length proteins and truncated isoforms encoded by both the wild-type and mutant alleles (126, 127). Structural and biophysical studies of the p53 tetramerization domain show that small changes in tetramer composition or stability can markedly alter transactivation potential and target gene specificity, supporting the idea that mixed tetramers will have distinct transcriptional outputs compared with homotetramers of wild-type or mutant p53. Consistent with this, experimental data demonstrate that Δ133p53 and Δ160p53-containing hetero-tetramers modulate promoter selectivity and can reprogram p53-dependent transcriptional profiles, either dampening canonical cell cycle arrest and apoptosis or biasing toward alternative, sometimes pro-tumorigenic, gene expression programs (82, 126, 128).

**Figure 3:**
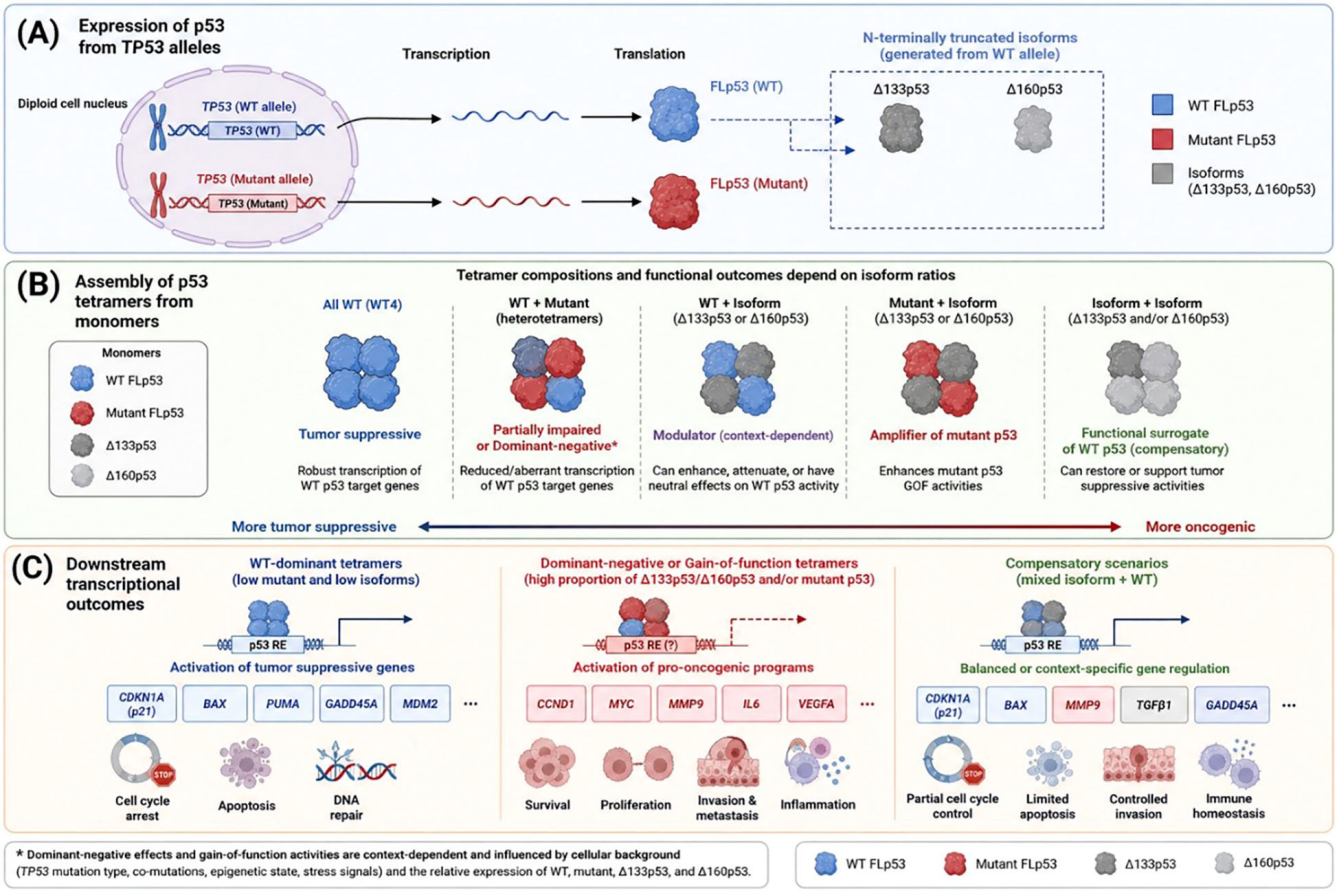
P53 protein isoforms in context of wild-type and mutated *TP53.* **(A)** In a diploid nucleus harboring a monoallelic TP53 mutation, the wild-type (WT) allele encodes full-length p53 (WT FLp53) as well as alternative N-terminally truncated isoforms, including Δ133p53 and Δ160p53, generated through alternative promoter usage and alternative translation initiation within the TP53 locus. The mutant TP53 allele typically carries missense alterations within the central DNA-binding domain and therefore still produces a structurally intact full-length mutant p53 protein (mutant FLp53), which can likewise give rise to short N-terminally truncated mutant isoforms via the same regulatory mechanisms. **(B)** Monomeric species (WT FLp53, mutant FLp53, Δ133p53, Δ160p53) co-assemble into tetramers whose composition and relative stoichiometry dictate functional output rather than absolute protein levels. WT homotetramers (all WT FLp53) are tumor suppressive and support robust transcription of canonical p53 target genes, whereas mixed WT–mutant tetramers show partially impaired or dominant-negative activity, leading to reduced or aberrant transcription of WT p53 targets. Incorporation of WT-derived truncated isoforms into heterotetramers can produce a spectrum of context-dependent effects: Δ133p53/Δ160p53 may act as modulators that attenuate, maintain, or redirect WT p53 activity; as amplifiers of mutant p53 gain-of-function, enhancing pro-oncogenic signaling; or, in some settings, as functional surrogates that compensate for impaired WT p53 signaling and partially restore tumor suppressive functions. **(C)** These distinct tetramer populations drive divergent transcriptional programs: WT-dominant tetramers favor activation of tumor-suppressive genes involved in cell-cycle arrest, apoptosis, and DNA repair; tetramers enriched in mutant FLp53 and/or high levels of Δ133p53/Δ160p53 promote activation of pro-survival, proliferative, invasive, and inflammatory gene networks; whereas mixed isoform–WT configurations can yield balanced or selectively re-routed p53 responses, including partial cell-cycle control, limited apoptosis, and context-specific regulation of invasion and immune homeostasis. Overall, the figure illustrates that the biological outcome of monoallelic TP53 mutation emerges from the dynamic, stoichiometry-dependent interplay between WT FLp53, mutant FLp53, and WT-derived truncated isoforms within mixed p53 tetramers. Note: The mutated TP53 allele can also undergo alternative splicing and promoter usage to generate mutated short isoforms (e.g., mutant Δ133p53). However, as their distinct structural and functional properties remain largely uncharacterized in the current literature, this figure focuses on the interactions of WT-derived alternative isoforms.

Moreover, in specific cellular and clinical contexts, p53 isoforms may act as functional surrogates of wild-type p53 rather than purely oncogenic drivers. For instance, in high-grade serous ovarian cancer, elevated Δ133p53 levels are strongly associated with prolonged survival even in the presence of dominant *TP53* mutations (112). Similarly, within hematological malignancies, high expression of short isoforms (Δ133/Δ160) in newly diagnosed multiple myeloma paradoxically correlates with a reduced risk of disease progression (72). These findings indicate that under defined stress conditions, illustrating that its biological role is not uniformly malignant (83). A schematic illustration of p53 isoforms in context of wild-type and mutated *TP53* is shown in **Figure 3**.

## 3 Detection of p53 protein isoforms

p53 isoforms, generated through alternative promoter usage, splicing, and translation initiation, require isoform-specific methods for accurate detection at mRNA and protein levels. Standard techniques leverage PCR for transcripts and immunoassays for proteins, often using specialized antibodies or primers to distinguish p53 variants. Moreover, most isoforms share large regions of sequence and are expressed at relatively low levels so that, their detection is technically challenging and requires carefully designed methods and controls (129). A summary of isoforms-specific detection methods is presented in **Table 1**.

**Table 1:** A summary of isoforms-specific detection methods.

Method	Short characteristics (material/mechanism)	Advantages in p53 isoform detection	Limitations
Nested RT-PCR / RT-qPCR	RNA-based assays using isoform-specific primers targeting alternative promoters, splice sites, or initiation regions; nested PCR increases sensitivity via two amplification rounds.	High sensitivity and specificity for low-abundance transcripts; enables discrimination and quantification of multiple TP53 isoforms.	Requires high-quality RNA; unreliable in FFPE samples; no information on protein level or PTMs.
Western blot (isoform-specific antibodies)	Protein-based method using antibodies against specific epitopes combined with molecular weight separation.	Detects protein isoforms and PTM-related shifts; widely used; semi-quantitative.	Requires sufficient sample; limited sensitivity; epitope masking/degradation; no single-cell resolution.
Capillary nanoimmunoassay (CNIA / Simple Western)	Automated capillary-based immunoassay using small sample volumes and isoform-specific antibodies.	High sensitivity with low input; suitable for limited samples; improved quantification; enables isoform classification.	Depends on antibody specificity; indirect detection; limited multiplexing.
LC-MS/MS with MIPs (targeted proteomics)	Mass spectrometry-based detection of isoform-specific peptides after trypsin digestion, supported by molecularly imprinted polymers.	High specificity; simultaneous quantification of isoforms (α, β, γ); antibody-independent.	Technically complex; requires specialized equipment; limited accessibility; challenges with very low-abundance isoforms.
Tagged p53 isoform expression (FLAG/HA/MYC)	Exogenous expression of tagged isoforms in cells, detected via tag-specific antibodies or MS.	Avoids low endogenous expression issues; no cross-reactivity; enables functional, localization, interaction, and PTM studies.	Artificial system; may not reflect physiological conditions; not suitable for direct patient samples.

### 3.1 mRNA-based assays

At the RNA level, isoform-specific nested RT-PCR (two amplification rounds) is the main method for quantitative TP53 isoform analysis. The first PCR targets a broad region; the second uses internal primers for high sensitivity/specificity in detecting low-abundance splice variants. RT-qPCR quantifies TP53 transcripts including TAp53, Δ40p53, Δ133p53, and α/β/γ isoforms using primers targeting alternative transcription initiation sites, internal promoters, or alternative splicing regions (117, 130, 131). These sensitive methods work well with high-quality RNA from cell lines or fresh-frozen tissue but their reliability decreases when dealing with fragmented RNA from formalin−fixed paraffin−embedded samples, and they can’t assess protein stability or post-translational modifications (PTM) directly (132).

### 3.2 Antibody-based assays

At the protein level, Western blotting with isoform-specific antibodies remains the main method to distinguish p53 isoforms, leveraging molecular weight separation and epitope mapping to differentiate full-length p53α from N-truncated Δ40p53, Δ133p53, Δ160p53, and C-spliced p53β variants (129). Key antibodies include: N-terminal DO-1/DO-7 (FLp53, p53β/γ-specific); PAb1801 (TA/Δ40); pantropic DO-11 and DO-12 (DBD epitope, all isoforms); KJC8 (p53β); MAP4.9/KJC133 (Δ133p53); KJC40 (Δ40p53); KJC160 (Δ160p53); and broad CM-1/SAPU (all isoforms via N/C/DBD epitopes) (72, 76). It enables semi-quantitative analysis of isoform levels and PTM-induced size shifts but requires sufficient amount of sample, lacks single-cell resolution, and faces epitope masking or degradation issues in tumors.

Rojas et al. used capillary nanoimmunoassay (Simple Western/CNIA) with DO1, DO11, A300-249A (α-specific), and KJC8 (β-specific) on CD138+ MM cells to detect isoforms from samples of limited size, classifying signals as long (TA/Δ40) or short (Δ133/Δ160) N-variants and α vs. β/γ C-variants, validated via isoform-transfected controls. qRT-PCR complemented this analysis at RNA level, revealing a distinct isoform profile in newly diagnosed MM and urging multi-isoform p53 assessments in cancer research (72, 133). **Figure 2** illustrates the mapping of antibodies specific to individual p53 isoforms.

### 3.3 Proteomics-based assay

Jiang et al. (134) developed a targeted proteomics assay combining molecularly imprinted polymers (MIPs) and Liquid Chromatography-Tandem Mass Spectrometry (LC-MS/MS) to simultaneously quantify C-terminal p53 isoforms α, β, and γ, hard to distinguish with conventional antibody-based assays.​ p53 isoforms were identified via proteotypic peptides: trypsin-digested samples yielded unique isoform-specific surrogate peptides sharing the N-terminal KPLDGEYFTLQ sequence but diverging at the C-terminus. These were validated in cell lysates by LC-MS/MS, confirming matching fragmentation spectra as reliable markers (134). The authors provided an alternative method to antibody-based workflows and opened new possibilities for quantitative isoform-level proteomics in both basic research and translational applications.

### 3.4 P53 tagging applications

Introducing expression vector-derived exogenous p53 isoforms fused to tags, such as FLAG, HA, and MYC, into cells offers a powerful approach to study and detect these proteins in controlled settings. This method bypasses challenges in detecting low-abundance endogenous isoforms, which often require complex antibodies or sensitive PCR techniques. Tagged isoforms enable precise detection via antibody-based methods such as Western blot, immunofluorescence, co-immunoprecipitation, or mass spectrometry avoiding cross-reactivity issues. Additionally, overexpression of tagged p53 isoforms in p53-null cells provides a clear experimental system by eliminating interference from endogenous p53 isoforms. It enables investigation of its subcellular localization, interactions with partner proteins, dominant-negative effects on other p53 isoforms, aggregation, and its functional impacts, as revealed by reporter gene assays and downstream gene expression profiling (102, 118, 126, 135). Finally, post-translational modifications, such as phosphorylation or ubiquitination, can be explicitly mapped to each isoform.

## 4 P53 protein isoforms in hematological malignancies

In comparison to solid tumors, mutations in the *TP53* gene are relatively rare in hematological cancers, however, aberrant expression patterns of p53 isoforms have been increasingly recognized as important factors in disease pathogenesis and prognosis. However, its clinical significance remains unclear.

### 4.1 Value of P53 isoform profiling with respect to: *TP53* mutation status, high-risk cytogenetics or MRD

p53 protein isoform profiling has emerging but still limited clinical utility in hematological malignancies, and its added value beyond *TP53* mutation status, high-risk cytogenetics and MRD remains largely investigational.

#### 4.1.1 Relationship to *TP53* mutation status

Current routine risk stratification in leukemias and myeloid neoplasms relies on *TP53* mutational analysis (including allelic state) rather than systematic isoform profiling. Multiple studies confirm that *TP53* mutations alone identify highly adverse subsets in AML, MDS and CLL, with strong prognostic and predictive impact on treatment resistance and overall survival (136, 137). However, p53 isoforms can modulate the functional consequences of a given *TP53* mutation, for example by dominant-negative or gain-of-function interactions within mixed oligomers (35, 83), suggesting that integrated assessment of sequence and isoform context may refine biological risk. A recent conceptual review argues that combining *TP53* mutation data with isoform information has the potential to improve functional classification of *TP53* lesions and to bridge the gap between genotype and phenotype (80). This incremental value is already evident in AML, where isoform profiles can distinguish outcomes between *FLT3-ITD* and *NPM1* mutational subsets (158), and in MM, where TAp53β/γ levels further stratify *TP53*-mutated cases (72). However, while these specific examples are promising, broad but clinical validation in hematology is still scarce.

#### 4.1.2 Interaction with high-risk cytogenetics

In myeloid malignancies, high-risk cytogenetics such as complex karyotype, del(17p), and chromosome 5 abnormalities are tightly linked to *TP53* disruption (138–141), yet current cytogenetic risk scores do not incorporate p53 isoform patterns. Strong nuclear p53 accumulation by immunohistochemistry, used as a surrogate of missense *TP53* mutation, independently predicts poor outcome in lower-risk del(5q) MDS and tracks expansion of *TP53*-mutated subclones (142, 143), but does not discriminate individual isoforms. Conceptually, distinct isoforms with different subcellular localization and transcriptional programs could help explain why patients with similar high-risk cytogenetics and *TP53* genotype show heterogeneous clinical trajectories. Specific evidence for this is emerging in multiple myeloma, where high levels of TAp53β/γ isoforms predict significantly worse survival even among patients already classified into the high-risk cytogenetic group, whereas elevated Δ133/Δ160 isoforms identify patients with a reduced risk of progression (72). Similarly, in pediatric B-cell ALL, the Δ133TP53 isoform is linked to the standard-risk ETV6::RUNX1 fusion, while TP53β expression specifically correlates with the high-risk KMT2A::AF4 fusion (144). Nonetheless, the available literature in hematologic cancers provides only isolated examples (e.g. MM, ALL) (72, 144) where defined isoform signatures correlate with survival, and these findings have not yet translated into modified cytogenetic risk categories.

#### 4.1.3 MRD setting and disease monitoring

Measurable residual disease assessment in leukemias currently relies on multiparameter flow cytometry, quantitative PCR or next-generation sequencing of leukemia-specific markers (145), not on p53 isoform assays. In MDS with del(5q), serial p53 immunostaining can identify expansion of TP53-mutated clones during progression (142), suggesting that protein-level readouts may complement MRD-like monitoring, although isoform-resolved methods were not applied. More broadly, the dynamic expression of p53 isoforms under genotoxic stress and therapy could, in principle, provide functional MRD information, but this concept remains hypothetical in hematology. At present there is no standardized, clinically approved MRD framework that incorporates p53 isoform profiling in AML, ALL, CLL or myeloma.

#### 4.1.4 Present and potential clinical value in hematological malignancies

In MM, an exploratory study showed that specific p53 isoform expression patterns predicted survival independently of conventional risk factors (72), providing the first direct evidence that isoform profiling can carry prognostic information in a hematologic cancer. It further suggests that integrating isoform expression with cytogenetic risk may refine prognostic stratification and identify novel subgroups within high-risk myeloma (72). Reviews of TP53 biology in hematologic malignancies emphasize that dysregulation of the p53 pathway is nearly universal and involves not only mutations and deletions but also alternative splicing, promoter usage and post-translational modification leading to diverse isoforms. Outside hematology, broader oncology data indicate that the clinical impact of p53 isoforms is highly tissue- and context-dependent, reinforcing the need for disease-specific validation before implementation.

Overall, p53 isoform profiling currently adds mainly exploratory mechanistic and prognostic insight on top of TP53 mutation status, high-risk cytogenetics and MRD, but it has not yet reached the level of evidence required for routine clinical decision-making in hematological malignancies.

### 4.2 Acute myeloid leukemia

AML is caused by the uncontrolled proliferation of clonal hematopoietic cells (146, 147). In AML, *TP53* alterations are relatively infrequent and closely associated with complex karyotype (16, 148–150). Furthermore, the frequency of mutations increases with age and in therapy-related AML (138). *TP53* status is a very important factor in risk classification (151), because mutations in this gene are associated with both poor prognosis and resistance to chemotherapy (152–155). In AML, altered expression of the p53 isoforms has been reported at both the mRNA and protein levels, despite mutations being found in only approximately 10% of cases (156). One of the studies conducted on 5 patients with AML showed, that during the induction therapy with idarubicine and cytarabine, there is a rapid increase in the concentration of the FLp53 isoforms, accompanied by a decrease in the level of the short isoforms (Δp53) and simultaneous activation of the expression of genes responsible for cell cycle arrest and apoptosis, including p21, BAX, PUMA, GADD45, MDM2, and subsequent cytopenia. To detect isoforms, researchers used NH_2_-terminal antibodies (157). Similarly, in another study analyzing 21 untreated AML patients, the upregulation of p53α and a downregulation of p53β/Ƴ were associated with increased sensitivity to *in vitro* applied valproic acid (VPA). Similar effects were observed in some patients treated with differentiation therapy using all-*trans* retinoic acid (ATRA) or theophylline (123). Moreover, it has been shown that higher levels of FLp53β and FLp53γ proteins are characteristic of the less differentiated AML (*p*-value < 0.005), suggesting that samples from patients at a more advanced stage of differentiation exhibit higher levels of more acidic (presumably phosphorylated and thus activated) p53 forms (123). Interestingly, in a study of 68 AML patients, p53 isoform expression has been found to correlate with specific genetic mutations. The authors reported that *FLT3* mutations were associated with expression of FLp53 protein isoforms and a poorer prognosis, whereas *NPM1* mutations correlated with expression of p53β and p53γ isoforms and better long-term survival (158).

Based on the clinical observations, there are possible molecular mechanisms underlying the isoform-related phenomena in AML.

### 4.3 Acute lymphoblastic leukemia

ALL is characterized by the neoplastic transformation and rapid proliferation of progenitor lymphoid cells in the bone marrow, bloodstream, and various tissues outside the marrow (159). There are three main types of ALL: T-ALL, B-ALL that cannot be further classified into a more specific subtype, and B-ALL with recurrent genetic defects, including *TP53* mutations (160, 161). The frequency of *TP53* mutations in ALL cases is approximately 16% (162), but the incidence range increases with age and among specific cytogenetic subgroups (162, 163). Moreover, *TP53* mutations are associated with early relapse and a poor prognosis (163–165). As mentioned above, p53 protein plays a significant role in ALL, particularly in B-cell precursor ALL (BCP-ALL). Oh et al. examined bone marrow samples from 50 pediatric patients with BCP-ALL (40 with *de novo* disease, 10 with relapse) who did not show *TP53* mutation, and from 4 healthy donors. The authors demonstrated that the expression patterns of p53 mRNA isoforms differ markedly between primary and relapse BCP-ALL. Regardless of the isoform, overall p53 mRNA levels were low in bone marrow from healthy donors. Primary BCP-ALL was characterized by strong expression of TAp53 (FLp53) in an active oligomeric conformation, and concurrent elevation of *CDKN1A* and *MDM2 levels*, suggesting activation of p53-dependent pathways. In contrast, relapsed BCP-ALL presented with elevated alternative p53 isoforms, such as Δ40p53, Δ133p53, and p53β, which exhibit an inactive quaternary conformation, together with increased *CDKN1A* but not *MDM2* levels, suggesting a change in the p53 isoform balance and a potentially altered or dysregulated p53 pathway response (166). Another group also analyzed profile of p53 isoforms in a larger pediatric B-cell cohort (n=100). They showed that full-length p53 isoforms were overexpressed in 52% of cases, whereas Δ40TP53, Δ133TP53, and TP53β were increased in 17%, 53%, and 6% of patients, respectively. Moreover, the researchers showed that Δ133TP53 expression exhibited a strong correlation with the occurrence of the *ETV6::RUNX1* gene fusion, which is associated with standard risk. In turn, occurrence of unfavorable *KMT2A::AF4* gene fusion showed an association with TP53β expression (144). These results suggest that Δ133TP53 may be a useful biomarker for identifying standard−risk patients, while TP53β may act as a marker of poor−risk ALL.

### 4.4 Multiple myeloma

MM is a clonal plasma cell neoplasm that constitutes over 10% of all hematologic cancers (167). Approximately 70% of MM diagnoses occur in patients over the age of 65 (168, 169). The Revised International Staging System (R-ISS) is used to stratify risk in patients with MM at diagnosis. It incorporates laboratory parameters such as β-2 microglobulin, albumin, and lactate dehydrogenase, and specific high-risk chromosomal changes: translocations t(4;14), t(14;16), or deletion of chromosome 17p (del17p) (170, 171), including *TP53 gene* that is located within the minimally deleted region on 17p13 (172). Moreover, it is known that among newly diagnosed MM patients, the deletion of chromosome del(17p) or *TP53* mutations are present in about 10% (173) and 3% (174) of patients, respectively, and their frequency is higher in more advanced stages of the disease (173–177). Rojas et al. described the profile of p53 isoforms in 156 patients ≤65 years of age with newly diagnosed MM who were treated as part of the PET HEMA/GEM2012 clinical trial with bortezomib, lenalidomide, and dexamethasone prior to autologous cell transplant. Fourteen of tested 147 patients (9.5%) had del17p. The qualitative evaluation of the CNIA (capillary nanoimmunoassay) assays revealed total p53 protein expression in 119 out of 156 (76%) MM samples, as detected with the DO−11 antibody, which binds to an epitope common to all p53 isoforms. The short p53 isoforms (Δ133/Δ160) were expressed in a minority of samples (18%), their high levels were associated with both progression and mortality risk reduction. Whereas overexpression of TA isoforms in particular TAp53β/Ƴ was related to a significantly worse prognosis, independently predicting both shortened time to progression and overall survival. Interestingly, this has been demonstrated not only in patients with standard cytogenetic risk but also in a high-risk cytogenetic group (72). Moreover, the researchers observed that TA isoforms were expressed at significantly higher levels in patients harboring *TP53* mutations than in those with a wild-type *TP53* gene. Currently, this is the only original work describing p53 isoforms in MM.

### 4.5 Chronic lymphocytic leukemia

CLL is an indolent cancer derived from dysfunctional mature B lymphocytes (178). Several prognostic factors are known to influence the course of the disease (179), however, clinical guidelines give the greatest importance to the predictive and prognostic value of the p53 pathway aberrations (180, 181). At the time of diagnosis, 4-10% of patients have p53 aberrations, including TP53 mutations and/or loss of the TP53 gene. Whereas, in refractory disease, abnormalities are detected in 42-45% of cases (22). Moreover, approximately 90% of CLL patients with del(17p) carry a *TP53* mutation, and ~ 60-70% of patients with *TP53* mutations also harbor del(17p), as detected by FISH (182). In addition, *TP53* mutation without a coexisting 17p deletion (sole *TP53* mutation) is identified in 5% of patients (183). *TP53* aberrations, even when detected subclonally in a small group of tumor cells, are associated with poor prognosis (184, 185). Thus, *TP53* status testing is indicated before starting any line of treatment (186). Sellmann et al. analyzed FLp53 (p53α) and its isoforms β and γ in 103 CLL patients and compared with B cells from 11 healthy donors. The FISH karyotype revealed 17p13 deletion in 11 cases; IGHV status was described as mutated, unmutated, or not done in 33, 30, 40 samples, respectively. The authors reported that the expression ratio of the studied isoforms was significantly altered in CLL samples compared to normal B cells. They observed highly predominant or comparable FLp53 (53 kDa) expression in CLL compared to the 46 kDa protein in samples taken from healthy donors, which corresponds to the TAp53β and γ isoforms. In contrast, normal B-cells were characterized by higher levels of TA53β-Ƴ compared to FLp53 isoforms. In an Western blot analysis, all samples with high p53 protein expression harbored a deletion of a single 17p13 allele. Additionally, the co-occurrence of 17p13 deletion and high levels of FLp53 protein was associated with a poorer prognosis (187). Overall, the altered expression pattern of p53 isoforms may impair the p53 response and contribute to the development of CLL.

### 4.6 Non-Hodgkin lymphomas

Data on the role of p53 isoforms in the biology of non-Hodgkin lymphomas are limited, and most results have been presented only as conference abstracts. Some studies show that *TP53* mutations and/or high p53 protein expression correlate with shorter OS and progression-free survival (PFS) in DLBCL patients treated with R-CHOP and other regimens (188–190). Similarly, in case of follicular lymphoma, *TP53* mutations correlate with unfavorable prognosis (191). A study in a mouse model revealed that animals overexpressing the Δ133p53 isoform, a murine homolog of human Δ133p53, exhibit suppressed apoptosis, increased cell proliferation, and develop a diverse range of aggressive tumors, including lymphoma (192).

### 4.7 Potential molecular mechanisms underlying p53 Isoform-Related phenomena in hematological malignancies

Apart from wild-type p53, the Δ133p53 is the best-characterized p53 isoform. By contrast, the roles of Δ160p53 and p53γ in regulating the p53 signaling pathway remain poorly understood. However, the clinical observations linking p53 isoforms to prognosis in hematological malignancies can be explained through several molecular mechanisms. Δ133p53 promotes DNA double-strand break repair by transcriptionally upregulating DNA repair genes including RAD51, RAD52 that enable leukemic cells to survive genotoxic stress (110, 193). This enhanced DNA repair capacity allows cells to tolerate chemotherapy-induced DNA damage rather than undergoing apoptosis finally causing resistance to the chemotherapy. Moreover, the association between specific p53 isoforms and poor prognosis in AML and MM may be mediated through activation of pro-inflammatory signaling cascades that create a favorable microenvironment for malignant cell survival. Δ133p53 activates pro-inflammatory signaling cascades in the bone marrow microenvironment such as the JAK-STAT3 signaling pathway through interleukin-6 -dependent mechanisms creating a positive feedback loop that promotes tumor cell survival, invasion, and metastasis via RhoA-ROCK pathway activation; and NF-κB target genes including IL-6, IL-8, and the anti-apoptotic protein Bcl-2, creating a chronic inflammatory state that supports malignant cell survival while inhibiting normal hematopoiesis (83, 118, 194). This inflammatory microenvironment confers competitive advantage to cells expressing aberrant p53 isoforms through paracrine effects on surrounding cells. In addition, Δ133p53β can regulate apoptosis by inhibiting the activity of an pro-apoptotic protein, RhoB, thereby enhancing cell survival (97). Moreover, Δ133p53 has the capacity to trigger signaling pathways of IFN-γ, while typically cytostatic, in the tumor microenvironment this may contribute to chronic inflammation and immune evasion mechanisms s (195–197). It has been reported that the Δ133p53β isoform is a key mediator in the promotion of cancer stem cell-like properties. It directly stimulates expression of key pluripotency factors such *SOX2*, *NANOG*, and *OCT3/4* (198), potentially increasing the risk of cancer recurrence. By extension, elevated Δ133p53 in hematological malignancies may shift the balance toward *NANOG* expression and stem cell maintenance, explaining the association with less differentiated leukemia phenotypes and poor long-term outcomes.

In contrast, our knowledge of Δ40p53 is more limited. However, it is known that In stem cell biology, Δ40p53 serves as a key regulator in pluripotent and progenitor cells, maintaining the balance between self-renewal and differentiation. Reduced levels of Δ40p53 result in the loss of pluripotency markers (including *NANOG* and *SSEA-1*), decreased proliferative capacity, and the emergence of cell cycle features characteristic of differentiated somatic cells. In contrast, overexpression of Δ40p53 sustains self-renewal and inhibits differentiation by influencing the activity of full-length p53 (FLp53) at critical pluripotency-associated targets, particularly NANOG and the insulin-like growth factor 1 receptor (IGF-1R) (199). In breast cancer cell lines, Δ40p53 is found in the same nuclear compartments as the pluripotency-associated transcription factors *SOX2, OCT4*, and *NANOG*, where it supports elevated expression of these stemness markers and augments both mammosphere formation and colony-forming capacity. Moreover, Δ40p53 correlates with reduced levels of the anti-stemness microRNAs miR-145 and miR-200, and with increased tumor burden, expanded microvascular networks, and heightened doxorubicin resistance *in vivo* (200). These findings support the conclusion that Δ40p53 acts as a key regulator of cancer stem cell maintenance and contributes to chemoresistance in breast cancer.

The amount of available data on the molecular mechanism of action and the role of the isoform Δ40p53. In embryonic stem cells, it regulates the switch from pluripotency to differentiation by tuning IGF signaling pathways, indicating that its mechanistic impact extends beyond classical tumor suppression to developmental and metabolic control (199). More recent work demonstrates that Δ40p53 can up-regulate netrin-1/UNC5B expression and modulate enhancer RNA (eRNA) transcription, suggesting that it acts partly through chromatin-level mechanisms and long-range gene regulation rather than simply behaving as a passive dominant-negative variant of full-length p53 (98).

These molecular mechanisms suggest several therapeutic strategies: (1) targeting Δ133p53-driven DNA repair through RAD51 inhibitors to resensitize chemotherapy-resistant cells; (2) blocking IL-6/JAK-STAT3 or NF-κB pathways to disrupt the pro-inflammatory microenvironment that supports isoform-driven malignant cell survival; (3) inhibiting stemness pathways through SOX2/NANOG targeting in tumors with elevated Δ133p53β; and (4) using IL-1β neutralizing antibodies to reduce inflammatory cytokine secretion in the bone marrow microenvironment.

### 4.8 Summary

Based on the analyzed data, we can hypothesize that the unifying prognostic model emerges across hematological malignancies. The ratio between full-length transcriptionally active p53 (FLp53/TAp53) and truncated/alternative isoforms (Δ40, Δ133, p53β/γ) may serve as a critical determinant of treatment response and disease outcome, though the direction of this correlation varies depending on disease stage and therapeutic context.

Across *de novo* AML, ALL and MM, specific p53 isoform patterns at diagnosis show consistent links with prognosis and treatment response. In AML, a shift toward dominance of full-length p53 with suppression of short/alternative isoforms under induction or differentiation-type therapy accompanies activation of canonical p53 targets and increased chemosensitivity, whereas particular baseline constellations (e.g. FLp53 with FLT3 mutations vs p53β/γ with NPM1 mutations) segregate poor- and good-risk groups. In pediatric BCP-ALL at first diagnosis, strong expression of TAp53/FLp53 with an active conformation and intact downstream signaling is typical and compatible with preserved p53 function, while, within *de novo* disease, Δ133TP53 associates with standard-risk ETV6::RUNX1-positive cases and TP53β with high-risk KMT2A::AF4-positive ALL, suggesting an isoform-based stratification of baseline genetic risk. In newly diagnosed MM treated with modern bortezomib/IMiD-based protocols, high levels of short Δ133/Δ160 isoforms paradoxically predict reduced risk of progression and death, whereas overexpression of TA isoforms, especially TAp53β/γ (often enriched in TP53-mutated cases), identifies patients with intrinsically poor prognosis and inferior survival despite intensive therapy.

In turn, during active chemotherapy, a striking reversal occurs: FLp53 rapidly increases while short isoforms decrease, coinciding with activation of cell cycle arrest and apoptotic genes. However, at relapse, the pattern inverts again—alternative isoforms (Δ40p53, Δ133p53, p53β) predominate, suggesting acquired resistance through isoform switching that circumvents conventional p53-mediated cell death pathways. This temporal evolution implies that initial isoform diversity provides therapeutic vulnerability, but selective pressure drives expansion of apoptosis-resistant isoform profiles.

The correlation between specific oncogenic drivers and isoform expression patterns (FLT3→FLp53→poor prognosis; NPM1→p53β/γ→favorable outcomes; ETV6::RUNX1→Δ133TP53; KMT2A::AF4→TP53β) indicates that distinct genetic lesions differentially affect p53 splicing, stability, or post-translational modifications. This suggests that isoform profiles are not merely biomarkers but mechanistically integrated into disease biology, potentially explaining why identical mutations yield heterogeneous clinical behaviors.

In CLL and MM, chromosomal deletion (del17p) combined with high expression of the remaining mutant TP53 allele creates the poorest prognostic subgroup. The retention and overexpression of this mutant protein, rather than simple haploinsufficiency, appears to drive aggressive disease. This occurs likely through dominant-negative effects that are further amplified when alternative regulatory isoforms—which could otherwise modulate this imbalance—are depleted. The data collectively suggest that therapies inducing differentiation (VPA, ATRA) may exert their effects partially through rebalancing the isoform repertoire toward configurations permissive for apoptosis. The observation that short isoforms in MM reduce progression risk despite being traditionally considered “inactive” challenges the binary functional/nonfunctional classification—these variants may retain context-dependent tumor-suppressive activities or modulate full-length p53 function through oligomerization dynamics. This tissue-specific divergence suggests that while Δ133p53-driven DNA repair and pro-inflammatory signaling may foster resistance in acute leukemias, in the unique genomic landscape of MM, these same isoforms may instead serve to attenuate the oncogenic ‘gain-of-function’ effects of mutant p53 or interfere with plasma cell-specific survival pathways.

This unified framework positions p53 isoform profiling as a potential precision oncology tool that integrates genetic context, treatment history, and disease stage to predict therapeutic vulnerability beyond conventional mutation analysis.

## 5 Therapeutic perspectives

Growing evidence positions p53 isoforms as clinical biomarkers and, in the longer term, potential therapeutic targets in hematological malignancies. No isoform-directed therapy is currently established in hematologic malignancies. Several strategies can be envisaged to therapeutically exploit isoform biology. These include, depending on the type of the disease: (i) restoring a favorable isoform balance (for example, by decreasing Δ40p53 or Δ133p53 while preserving TAp53α function), (ii) selectively inhibiting N−terminally truncated isoforms with dominant−negative or pro−survival functions, and (iii) stabilizing C−terminally spliced isoforms that enhance senescence or apoptosis.

The practical implementation of these strategies requires molecular technologies capable of modulating TP53 splicing or isoform expression at the cellular level. In principle, antisense oligonucleotides, splice−switching oligonucleotides, or small molecules that modulate splicing decisions at intron 2 and intron 9 could shift the TP53 transcriptome toward isoforms that promote tumor suppression. Such technologies are increasingly feasible, as comparable approaches are already in clinical use for other splice−dependent diseases, suggesting that similar technologies might be adapted to re−program p53 isoform expression in leukemia or myeloma. For example, nusinersen (Spinraza), a splice-switching antisense oligonucleotide that alters SMN2 pre-mRNA splicing to include exon 7, was approved by the U.S. Food and Drug Administration in December 2016 for the treatment of spinal muscular atrophy (SMA) across all disease types and age groups (201, 202). Similarly, eteplirsen, a phosphorodiamidate morpholino oligomer that induces DMD exon 51 skipping, received conditional FDA approval in 2016 for Duchenne muscular dystrophy (DMD), although its efficacy remains limited and subsequent generations of exon-skipping antisense oligonucleotides targeting alternative sites show significantly improved potency (203, 204). These clinical successes establish the feasibility of antisense-mediated splice modulation as a therapeutic modality and provide a translational roadmap for targeting TP53 splicing aberrations in hematological malignancies.

Beyond direct isoform modulation, a more immediately translatable approach is patient stratification based on isoform profiles. The clinical data reviewed in this manuscript demonstrate that specific isoform patterns correlate with distinct outcomes across hematological malignancies: in AML, *FLT3* mutations associate with elevated FLp53 expression and poorer prognosis, while *NPM1* mutations correlate with increased p53β and p53γ levels and improved long-term survival; in ALL, Δ133TP53 expression strongly correlates with favorable *ETV6::RUNX1* fusion and standard-risk disease, whereas TP53β associates with high-risk *KMT2A::AF4* fusion; in MM, overexpression of TAp53β and TAp53γ independently predicts both shortened time to progression and overall survival; and in CLL, elevated p53 isoform expression correlates with chemotherapy resistance and increased transformation risk to Richter syndrome. These observations suggest that comprehensive isoform profiling could refine risk stratification beyond TP53 mutation status and cytogenetics alone, and could identify subgroups of patients who might benefit from treatment intensification, modified chemotherapy schedules, or novel targeted approaches based on their isoform landscape. A summary of specific isoforms and their role in the course of particular hematological diseases is presented in **Table 2**.

**Table 2:** A summary of specific isoforms and their role in the course of particular hematological diseases.

Disease	p53 isoform(s) involved	Impact on treatment and biology	Impact on prognosis	References
Acute myeloid leukemia (AML)	FLp53 (p53α), p53β, p53γ, short Δp53 isoforms	Higher FLp53/lower Δp53 or p53β/γ increases chemosensitivity	**Favorable:** NPM1 mutations + p53β/γ. **Poor:** FLT3-ITD mutations + FLp53.	(123, 157, 158)
Acute lymphoblastic leukemia (ALL)	TAp53 (FLp53), Δ40p53, Δ133p53, p53β, Δ40TP53, Δ133TP53, TP53β	**BCP-ALL:** Δ40/Δ133/p53β-rich patterns lead to chemoresistance and relapse B-cell ALL: Δ133TP53 correlates with *ETV6::RUNX1* gene fusion TP53β correlates with *KMT2A::AF4* gene fusion	**BCP-ALL:** **Standard:** TAp53-rich profiles. **High Risk:** Alternative isoforms (Δ40p53, Δ133p53, and p53β) **B-cell ALL:** **Standard**: Δ133TP53 correlated with *ETV6::RUNX1* gene fusion **Poor**: TP53β correlated with *KMT2A::AF4* gene fusion	(144, 166)
Multiple myeloma (MM)	Short N-terminally truncated isoforms (Δ133/Δ160), TAp53β/γ	High Δ133/Δ160 levels reduces progression risk during therapy High TAp53β/γ levels correlates with poor prognosis	**Favorable:** High Δ133/Δ160 levels **Poor:** High TAp53β/γ levels	(72)
Chronic lymphocytic leukemia (CLL)	FLp53 (p53α), TAp53β/γ and other p53 isoforms	Disturbed isoform balance causes resistance to chemoimmunotherapy	**Poor:** High FLp53 (with 17p deletion) and high total isoform levels	(187)

B-Cell Precursor Acute Lymphoblastic Leukemia (BCP ALL).

Finally, prospective clinical validation of isoform-based risk stratification will require systematic mapping of p53 isoform landscapes in large, molecularly annotated cohorts of AML, ALL, MM, and CLL to identify isoform signatures that predict benefit from specific drug classes (hypomethylating agents, venetoclax−based combinations, proteasome inhibitors or Bruton’s tyrosine kinase inhibitors). Such isoform-derived signatures should be validated in independent cohorts and integrated with established biomarkers such as TP53 mutations, complex karyotype, and minimal residual disease status. Such signatures could be integrated into future risk−stratification algorithms, complementing TP53 mutation status and cytogenetic risk, and could guide the development of truly isoform−directed therapies in hematology.

## 6 Knowledge gaps and future research directions

Overall, the clinical literature on p53 isoforms in hematological malignancies remains limited by small cohort sizes, heterogeneous assay platforms, incomplete alignment between transcript- and protein-level measurements, and inconsistent use of clinical endpoints.

To advance this field of research, standardization of p53 isoform nomenclature and harmonization of mRNA and protein assays are essential. Studies should systematically map p53 isoform expression in hematological malignancies and various treatment contexts, link isoform patterns to clinically relevant parameters, and determine their functional impact during disease progression.

Future work should integrate high-resolution isoform quantification (isoform-specific quantitative reverse-transcription polymerase chain reaction, multiplex antibody panels, capillary nanoimmunoassays, and targeted liquid chromatography–tandem mass spectrometry) with functional assays in primary tumor cells and *in vivo* models to dissect isoform-specific roles in DNA-damage responses, microenvironmental interactions, and immune evasion. In parallel, translational research should evaluate antisense and splice-modulating strategies to shift the TP53 transcriptome toward tumor-suppressive isoforms and test rational drug combinations that exploit isoform-dependent vulnerabilities, paving the way for truly isoform-directed therapies.

## 7 Conclusions

In summary, p53 isoforms add an important layer of complexity to TP53 biology in hematological malignancies. The available evidence supports their biological relevance and suggests possible associations with disease behavior, treatment response, and outcome. However, the field is still constrained by limited cohort sizes, methodological heterogeneity, and the lack of standardized clinically validated assays. Future studies should focus on harmonized isoform detection, integration with TP53 mutational and cytogenetic status, and prospective evaluation in well-annotated patient cohorts.
